# The Associations of AI Literacy, AI Self-Efficacy and AI Attitudes Among Nursing Students: A Cross-Sectional Path Analysis

**DOI:** 10.3390/nursrep16080292

**Published:** 2026-08-21

**Authors:** Shinhi Han, Hee Sun Kang, Philip Gimber, Sunghyun Lim

**Affiliations:** 1 Department of Health Sciences, LaGuardia Community College, City University of New York, Long Island City, NY 11101, USA; shan@lagcc.cuny.edu (S.H.); pgimber@lagcc.cuny.edu (P.G.); 2 Red Cross College of Nursing, Chung-Ang University, Seoul 06974, Republic of Korea; goodcare@cau.ac.kr; 3 Critical Care Medicine, Columbia University Irving Medical Center, New York, NY 10032, USA

**Keywords:** GenAI, nursing students, Social Cognitive Theory, AI literacy, AI self-efficacy, AI attitudes

## Abstract

**Background**: Generative Artificial Intelligence (GenAI) is increasingly integrated into nursing education, yet structured AI literacy training and ethical guidance remain limited. Consequently, nursing students often rely on informal learning, resulting in variability in AI readiness, confidence, and responsible use. **Aims**: This study was conducted to examine (1) whether AI literacy was positively associated with AI self-efficacy and AI attitudes and (2) whether AI self-efficacy mediated the relationship between AI literacy and AI attitudes. **Methods**: A cross-sectional survey using convenience sampling was conducted with 100 prelicensure nursing students in New York City. Data were collected using the AI Literacy Scale (AILS), AI Self-Efficacy Scale (AISES), and Generative AI Attitude Scale (GAIAS). Correlation and path analyses were performed using SPSS and Amos 30.0. **Results**: The participants had a mean age of 30.25 years, and 71% were women. AI literacy and AI self-efficacy were both positively associated with AI attitudes (all *p* < 0.001). Path analysis showed that AI literacy significantly predicted AI self-efficacy (β = 0.39, *p* < 0.001) and AI attitudes (β = 0.28, *p* = 0.003). AI self-efficacy significantly predicted AI attitudes (β = 0.31, *p* = 0.001) and partially mediated the relationship between AI literacy and AI attitudes. **Conclusions**: AI self-efficacy partially mediated the relationship between AI literacy and AI attitudes. Nursing curricula may benefit from structured AI education that integrates guided GenAI practice, case-based learning, and faculty feedback. Such educational frameworks warrant further empirical investigation regarding their potential to foster AI literacy, AI self-efficacy, and positive attitudes toward responsible AI integration, particularly through longitudinal studies assessing subsequent behavioral outcomes.

## 1. Introduction

### 1.1. Background

Generative Artificial Intelligence (GenAI) is driving a paradigm shift in nursing education and healthcare, transforming how students learn, access information, and prepare for clinical practice [1]. Nursing students increasingly use GenAI tools to support academic learning, synthesize complex medical–surgical concepts, and enhance clinical preparation [2,3]. Despite the rapid adoption of GenAI in nursing education, structured AI literacy training, clear ethical guidelines, and discipline-specific instructional strategies have not yet been systematically integrated into nursing curricula [4]. Consequently, many students rely on self-directed, peer-led, or informal learning experiences to develop AI-related competencies. Such unstructured approaches may result in considerable variability in students’ readiness, confidence, and ability to use AI responsibly and effectively in academic and clinical settings [5,6]. Understanding students’ preparedness and educational needs is therefore essential for developing safe, effective, and pedagogically sound AI-integrated nursing education [3,4].

Current evidence suggests that although nursing students demonstrate strong interest in AI technologies and digital innovation, their levels of AI literacy remain inconsistent [3,4]. Deficiencies are particularly evident in areas requiring higher-order judgment, including ethical decision-making, recognition of algorithmic bias, and critical evaluation of AI-generated information [4,7]. When AI competencies are acquired primarily through informal and unverified sources, students may develop fragmented or inaccurate understandings of the capabilities and limitations of AI, potentially compromising their ability to critically appraise AI-generated content and apply these technologies appropriately in patient care contexts.

AI literacy has emerged as a foundational competency for the effective and responsible use of AI technologies. Students with higher levels of AI literacy are more likely to understand the capabilities, limitations, and ethical implications of AI systems, enabling them to critically evaluate AI-generated outputs, identify potential biases, and engage with GenAI tools more effectively [8,9,10]. Greater AI literacy has also been associated with increased confidence in using AI technologies and more favorable perceptions of their usefulness in learning environments [10,11,12,13,14]. Conversely, limited AI literacy may contribute to anxiety, skepticism, and concerns regarding misinformation, ethical misuse, and overreliance on AI-generated content [11,15,16,17].

Emerging evidence further suggests that AI self-efficacy plays a critical role in shaping students’ engagement with AI technologies. Individuals who feel confident in their ability to use AI tools are more likely to integrate them into academic learning, clinical preparation, and decision-making processes [18]. Positive attitudes toward AI have likewise been associated with greater technology acceptance, behavioral intentions to use AI, and self-efficacy across previous studies [10,13,18]. Moreover, students’ attitudes toward AI appear to be influenced by their level of AI literacy, with greater knowledge and understanding fostering more balanced, informed, and constructive perceptions of AI technologies [11,17]. Collectively, these findings highlight the importance of structured educational guidance, ethical frameworks, and intentional curricular integration to support responsible and confident AI engagement among nursing students [3,4,19].

Research in nursing education has largely focused on evaluating general knowledge and intention, assessing overall AI readiness, or identifying direct correlations between AI use and students’ attitudes and perceptions [3,4,7,10,11,13,14,17,20]. However, a critical knowledge gap remains regarding the specific cognitive–belief mechanisms through which foundational AI literacy translates into favorable attitudinal outcomes. Existing nursing research has primarily examined direct relationships between AI literacy or related competencies and attitudes, readiness, and intentions to use AI, with comparatively limited attention to the psychological mechanisms underlying these associations. This gap is particularly relevant because AI attitudes may represent an important proximal outcome associated with students’ intentions to engage with AI and, potentially, their use of these technologies [13]. Accordingly, examining AI self-efficacy as a potential explanatory mechanism may provide a more nuanced understanding of how AI literacy is associated with students’ AI attitudes.

**Figure 1:**
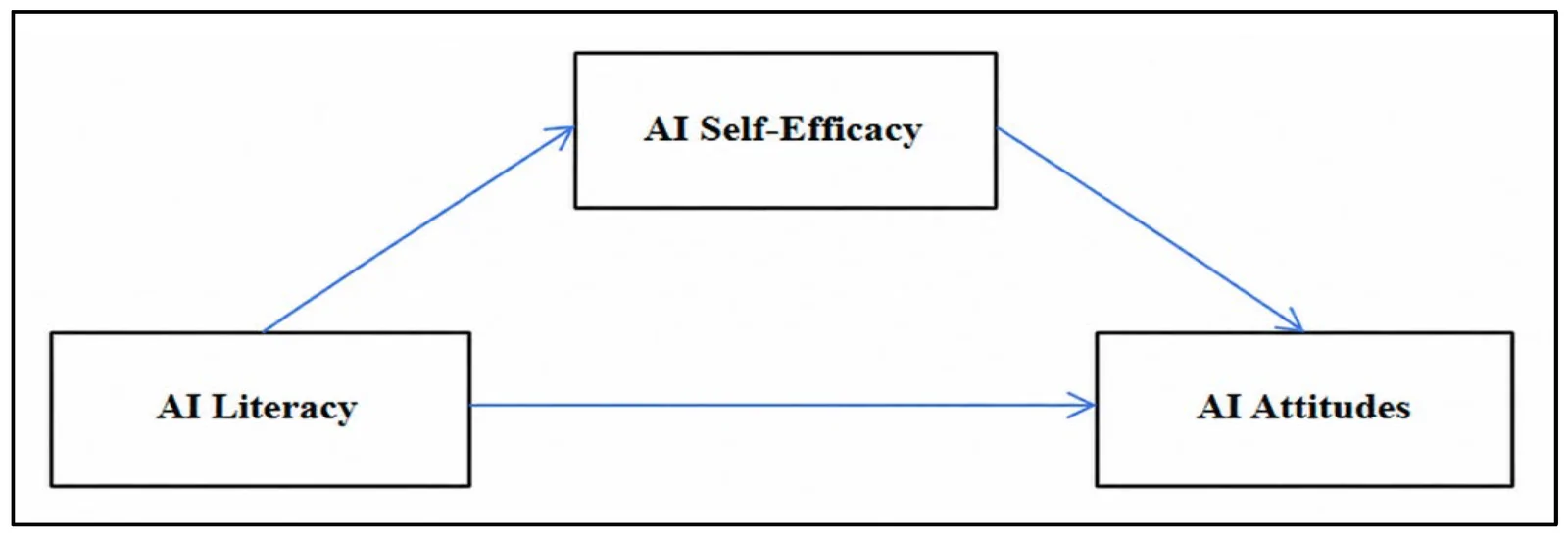
Conceptual model. AI: artificial intelligence.

To advance research on AI in nursing beyond descriptive findings and simple bivariate associations, this study examined a potential explanatory pathway linking AI literacy, AI self-efficacy, and AI attitudes (Figure 1). According to Social Cognitive Theory (SCT), individuals do not act solely on external knowledge; rather, their self-evaluations of capability play a critical role in shaping affective orientations and behavioral intentions. AI self-efficacy was selected as the primary mediator because SCT positions self-efficacy as a pivotal cognitive mechanism through which knowledge and domain-specific competencies translate into attitudinal dispositions.

### 1.2. Theoretical Framework and Conceptual Model

This study is grounded in Albert Bandura’s Social Cognitive Theory (SCT), which posits that human behavior and learning emerge from dynamic interactions among cognitive factors, self-referent personal beliefs, and affective outcomes [21,22]. SCT specifically supports the hypothesized mediation model by postulating that AI literacy does not automatically generate positive attitudinal dispositions; rather, knowledge must first be cognitively processed and integrated into self-referent beliefs regarding personal mastery (AI self-efficacy).

Within this framework, a sequential cognitive–belief–affective pathway is operationalized, in which AI literacy (cognitive factor) represents students’ perceived knowledge, structural understanding, and critical appraisal of GenAI mechanisms; AI self-efficacy (personal belief mediator) captures students’ internal confidence in their ability to successfully execute tasks and troubleshoot using GenAI tools responsibly; and AI attitudes (affective outcome) reflect students’ evaluative perceptions regarding the utility, engagement, safety, and relevance of GenAI in educational and clinical settings. Although Bandura’s classical formulation of SCT highlights triadic reciprocal determinism—where cognitive, behavioral, and environmental influences operate bidirectionally over time—our conceptual model intentionally operationalizes a single directional path (AI literacy → AI self-efficacy → AI attitudes).

### 1.3. Purpose and Hypotheses

This study was conducted to examine (1) whether AI literacy was positively associated with AI self-efficacy and AI attitudes and (2) whether AI self-efficacy mediated the relationship between AI literacy and AI attitudes. The hypotheses of this study are as follows:

**H1.**  *AI literacy is positively associated with AI self-efficacy*.

**H2.**  *AI self-efficacy is positively associated with AI attitudes*.

**H3.**  *AI literacy is positively associated with AI attitudes*.

**H4.**  *AI self-efficacy mediates the relationship between AI literacy and AI attitudes*.

## 2. Methods

### 2.1. Study Design

A descriptive, cross-sectional quantitative survey design was employed to evaluate the direct and indirect relationships between artificial intelligence (AI) literacy, AI self-efficacy, and AI attitudes among prelicensure nursing students.

### 2.2. Participants and Settings

A convenience sampling strategy was used to recruit undergraduate nursing students from a public community college nursing program in New York City. The inclusion criteria required participants to be (1) actively enrolled in nursing clinical tracks within the nursing program and (2) willing to provide voluntary informed consent. Students not actively enrolled in the nursing curriculum were excluded. The study took place in a program in which GenAI had not yet been formally integrated into the curriculum, and the students had received no formal GenAI instruction prior to participating in the study.

A total of 110 participants initially completed the survey. Following a systematic data-cleaning process, 10 incomplete responses (defined as questionnaires with incomplete data for one or more study measures) were excluded, resulting in a final analytical sample of N = 100. This sample size meets the established methodological threshold of a minimum of 100 responses required to yield stable and valid maximum likelihood estimations in structural equation modeling (SEM) path analyses [23,24].

### 2.3. Procedures and Data Collection

This study was approved by the institutional review board at the first author’s college (2025-0048-LCC). Following IRB approval and authorization from the department chair, recruitment flyers containing a secure hyperlink and QR code to the study survey were distributed at the study sites. The flyers provided eligible students with information about the study and instructions for accessing the survey. Students who were interested in participating independently accessed the secure, web-based survey hosted on the Qualtrics platform using the hyperlink or QR code provided on the recruitment flyer. Participation was voluntary, and students self-selected to participate in the study without direct enrollment by the researchers. Upon accessing the survey, prospective participants were presented with an electronic informed consent form outlining the study’s purpose, procedures, and confidentiality protections, in addition to the voluntary nature of participation and their right to withdraw. Only individuals who provided electronic informed consent were permitted to proceed to the demographic questionnaire and validated psychometric instruments. Of the 110 students who accessed and initiated the survey, 100 completed it, yielding a completion rate of 91% (100/110).

### 2.4. Measures

Permission to use the measurement instruments was obtained from the original developers prior to data collection.

#### 2.4.1. Artificial Intelligence Literacy (AI Literacy)

AI literacy was measured using the 12-item Artificial Intelligence Literacy Scale (AILS) developed by Wang et al. [25]. The AILS assesses four domains of AI literacy: awareness, usage, evaluation, and ethics. Each item is rated on a 7-point Likert scale ranging from 1 (strongly disagree) to 7 (strongly agree). Three negatively worded items (items 2, 5, and 11) were reverse-scored prior to analysis. A total score was obtained by summing the item scores as recommended by Wang et al. [25], with higher total scores indicating greater AI literacy. At the time of its development, the instrument demonstrated content validity and construct validity through confirmatory factor analysis. Cronbach’s alpha was 0.83 in the original validation study and 0.80 in the present study.

#### 2.4.2. Artificial Intelligence Self-Efficacy

AI self-efficacy was measured using the 22-item Artificial Intelligence Self-Efficacy Scale (AISES) developed by Wang and Chuang [26]. The AISES assesses four domains of AI self-efficacy: conceptual understanding, practical application, critical evaluation, and ethical and responsible use. All items are rated on a 7-point Likert scale ranging from 1 (strongly disagree) to 7 (strongly agree). A total score was obtained by summing the item scores, with higher scores indicating greater AI self-efficacy. At the time of its development, the instrument demonstrated content validity and construct validity through confirmatory factor analysis. Cronbach’s alpha was 0.96 in the original validation study and 0.94 in the present study.

#### 2.4.3. Artificial Intelligence Attitude

AI attitudes were assessed using the 13-item Generative Artificial Intelligence Attitude Scale (GAIAS) developed by Marengo et al. [27]. The GAIAS measures both positive (8 items) and negative (5 items) attitudes towards AI. Negative attitudes assess concerns related to future impact, the learning process, and reliability and trust.

Responses are rated on a 5-point Likert scale ranging from 1 (strongly disagree) to 5 (strongly agree). Five negatively worded items were reverse-scored prior to analysis. A total score was obtained by summing the item scores, with higher total scores indicating more positive AI attitudes. At the time of its development, the instrument demonstrated content validity and construct validity through confirmatory factor analysis. Cronbach’s alpha was 0.85 in the original validation study and 0.83 in the present study.

### 2.5. Ethical Considerations

This study was approved and overseen by the Institutional Review Board of the City University of New York (CUNY). Data security measures were implemented to ensure anonymity; no personally identifiable information (PII) or IP addresses were collected during the web survey. Participants were explicitly informed that their participation or choice to withdraw would have no bearing on their academic standing, grades, or relationship with the college.

### 2.6. Data Analysis

The data were analyzed using SPSS version 30.0 and AMOS version 30.0 (IBM Corp., Armonk, NY, USA). Descriptive statistics (means, standard deviations, frequencies, and percentages) were computed to summarize participants’ characteristics and the study variables. Normality of continuous variables was assessed using skewness and kurtosis. Pearson’s correlation coefficients were calculated to examine the relationships among the three study variables. Missing data and the assumptions underlying structural equation modeling (SEM) were examined prior to the analysis. The extent of and patterns in missing data were assessed by examining the percentage of missing values for each variable. The amount of missing data was minimal, and no systematic pattern of missingness was identified. Therefore, missing data were handled using full information maximum likelihood (FIML) estimation.

To test the hypothesized mediation model, observed-variable path analysis was performed using AMOS 30.0. Bootstrapping is the preferred method for testing indirect effects [28]. Thus, the indirect effect of the independent variable on the dependent variable through the mediator was estimated using a bootstrapping approach with 5000 resamples, considering a 95% bias-corrected confidence interval (CI), with significance indicated when the interval did not include zero. In addition, a bootstrap Monte Carlo power analysis for the mediation model was conducted using R version 4.2.2 based on the effect size and model parameters observed in the present study. The analysis involved 1000 simulated datasets, each consisting of 100 participants, with 5000 bootstrap samples. The estimated statistical power was 92.5%, suggesting that the sample size was adequate. All path coefficients are reported as standardized estimates. All statistical tests were two-tailed, with the significance level set at *p* < 0.05.

## 3. Results

### 3.1. Participant Characteristics

The participants’ mean age was 30.25 years (standard deviation [*SD*] = 7.84, range 19–48), and 71.0% were women. Most participants were in the first semester (36.0%) or the second semester (44.0%) and had a Grade Point Average (GPA) above 3.0.

Most participants reported that their computer skills were at an intermediate (64.0%) or advanced level (23.0%). GenAI use for academic purposes was reported as frequent (weekly, 27.0%) or very frequent (multiple times per week, 39.0%). The characteristics of the participants are summarized in Table 1.

### 3.2. Descriptive Statistics of Study Variables and Correlations

Table 2 presents the descriptive statistics and the bivariate correlations among the study variables. AI literacy was significantly positively associated with AI self-efficacy (*r* = 0.42, *p* < 0.001) and AI attitudes (*r* = 0.40, *p* < 0.001). AI self-efficacy was significantly positively associated with AI attitudes (*r* = 0.42, *p* < 0.001).

### 3.3. Path Coefficients and Mediating Effect of AI Self-Efficacy

Prior to conducting the path analysis, the SEM assumptions were evaluated. Skewness (−0.910 to −0.069) and kurtosis (0.322 to 3.230) were within the recommended thresholds (|skewness| < 2.0; |kurtosis| < 7.0), indicating acceptable normality, and both variance inflation factors (VIFs) were 1.179, below the recommended cutoff of 5.0, indicating no evidence of multicollinearity. We conducted Harman’s single-factor test. The first unrotated factor accounted for 33.43% of the total variance, which is below the commonly accepted threshold of 50%, suggesting that common method bias is unlikely to substantially affect the findings. Since the hypothesized model was only identified (χ^2^ = 0.000, df = 0), model fit indices were not interpreted.

**Figure 2:**
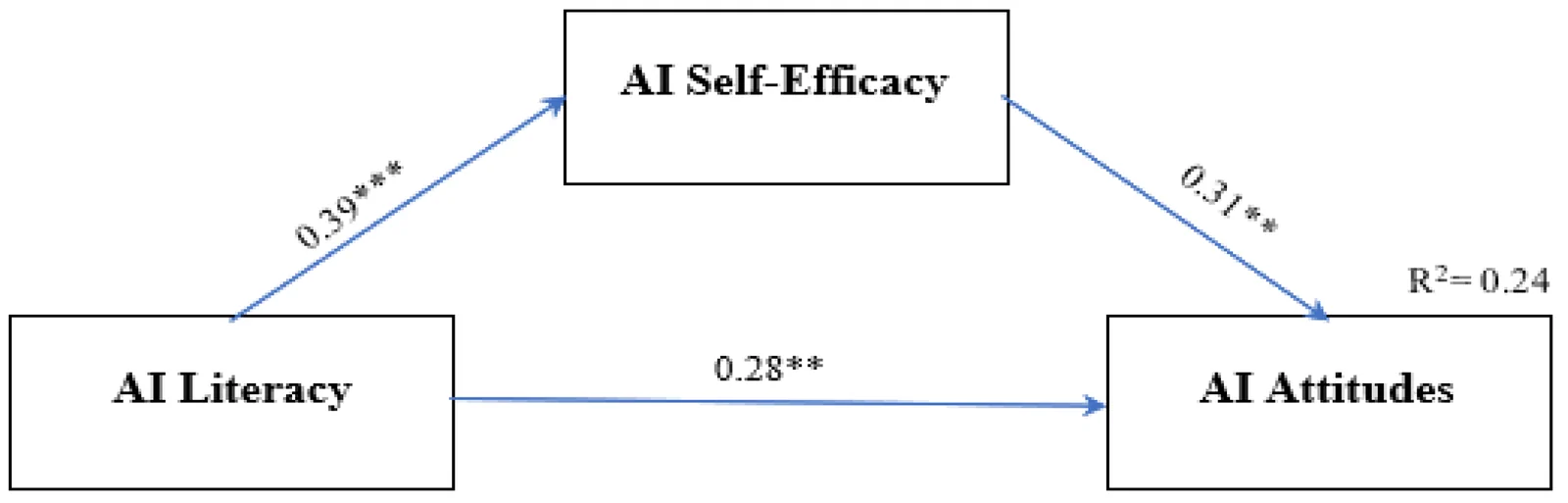
Path model of AI attitudes with standardized path coefficients (β). Note. AI = artificial intelligence. ** *p* < 0.01 *** *p* < 0.001.

The results of the path analysis (Table 3) indicated that AI literacy was positively associated with AI self-efficacy (*β* = 0.39, *p* < 0.001; H1) and AI self-efficacy was positively associated with AI attitudes (*β* = 0.43, *p* < 0.001; H2). In addition, AI literacy was positively associated with AI attitudes (*β* = 0.28, *p* = 0.003; H3). The indirect association of AI literacy with AI attitudes through AI self-efficacy was significant (B = 0.07; 95% CI [0.03, 0.15]; H4), as the confidence interval did not include zero, supporting the mediating role of AI self-efficacy. Therefore, H1, H2, H3, and H4 were supported. Overall, the model explained 24% of the variance in AI attitudes. A path model with the standardized coefficients and the squared multiple correlation (R^2^) is presented in Figure 2.

## 4. Discussion

We found that AI literacy was positively associated with AI self-efficacy and AI attitudes and that AI self-efficacy partially mediated the relationship between AI literacy and AI attitudes. These findings suggest that AI literacy, AI self-efficacy, and AI attitudes are closely interrelated and may collectively help prepare nursing students for the paradigm shift in nursing education and healthcare driven by GenAI.

The positive association between AI literacy and AI self-efficacy is consistent with previous studies demonstrating that greater AI knowledge is associated with higher confidence in using AI technologies [8,9]. Similar findings have been reported across healthcare and higher education settings, where individuals with stronger AI-related knowledge perceive themselves as more capable of using GenAI tools effectively [8,9,13,15,29]. These findings are particularly relevant because they may provide insight into major concerns identified in previous studies regarding AI use, including ethical considerations, limitations, bias, and the reliability of AI-generated information [3,4,15]. As students improve their AI literacy, they may be better equipped to evaluate AI-generated outputs critically, recognize potential biases and inaccuracies, and understand the appropriate application of AI in healthcare practice [11,15,16,17]. Boztepe et al. [30] also highlighted that students’ ability to use AI and analyze it through a critical filter is directly associated with their readiness for AI. Furthermore, a deeper understanding of AI concepts and limitations may contribute to greater confidence in students’ ability to use these technologies effectively and responsibly. This interpretation may be consistent with Social Cognitive Theory, which posits that knowledge contributes to self-efficacy by strengthening individuals’ perceived capability to perform a given task successfully [21,22].

In our study, AI self-efficacy was positively associated with AI attitudes. Similarly, previous studies have shown that individuals with greater confidence in using AI tend to hold more favorable attitudes toward these technologies [10,11,12]. These findings suggest that strengthening AI self-efficacy may be a potential educational approach to explore in future intervention studies examining attitudes toward GenAI among nursing students. Consistent with recent evidence linking AI literacy to attitudes toward GenAI, our findings also demonstrated a positive association between AI literacy and AI attitudes. Sun et al. [13] similarly reported associations among AI literacy, attitudes, and behavioral intentions toward GenAI, with behavioral intentions directly associated with actual GenAI use. Given the increasing prominence and accessibility of GenAI in higher education, general AI literacy and AI self-efficacy may represent foundational competencies that shape nursing students’ attitudes toward and engagement with these technologies. Together, these findings suggest that AI attitudes may have broader behavioral relevance, although longitudinal research is needed to clarify the relationships among attitudes, behavioral intentions, and actual GenAI use.

Building on these associations, our study also revealed the partial mediating effect of AI self-efficacy on the relationship between AI literacy and AI attitudes. This finding suggests that AI knowledge alone may not be sufficient to foster positive attitudes toward AI. Rather, students must also develop confidence in their ability to apply AI effectively and responsibly. The present findings are generally consistent with those of Asio [18], who also identified AI self-efficacy as a mediator among AI-related variables. Although Asio [18] proposed a different directional pathway, both studies emphasize the central role of self-efficacy in linking AI-related knowledge and perceptions. However, both studies employed cross-sectional designs, and thus causal relationships cannot be established. Future longitudinal studies are needed to clarify the temporal relationships among AI literacy, AI self-efficacy, and AI attitudes.

In addition, although the model explained 24% of the variance in AI attitudes, this finding should be interpreted with caution, as other factors not included in the model may also contribute to nursing students’ attitudes toward AI. Boztepe et al. [30] emphasized that nursing students’ attitudes toward and readiness for AI adoption may be shaped not only by AI-related knowledge but also by experience-based engagement with AI technologies and a strong ethical foundation fostered through ethics education. Taken together, these findings suggest that AI attitudes are likely to be influenced by multiple factors beyond those examined in the present model. Further studies exploring factors influencing AI attitudes are warranted.

The present study has meaningful implications for nursing education because it can inform the development of future content and priorities in nursing curricula. First, nursing curricula should incorporate structured AI education that develops students’ foundational AI literacy. Second, educational strategies should extend beyond increasing AI knowledge to intentionally strengthen AI self-efficacy. Guided practice with GenAI tools, simulation exercises, case-based learning, and faculty mentorship can provide meaningful opportunities for students to apply GenAI in real clinical and educational contexts while receiving constructive feedback. These mastery experiences, as described by Social Cognitive Theory, may strengthen students’ confidence in using AI effectively and responsibly. Because AI self-efficacy statistically accounted for part of the association between AI literacy and AI attitudes in the present model, future intervention studies should examine whether educational strategies targeting AI literacy and AI self-efficacy result in more positive attitudes toward AI. Overall, the present findings suggest that effective AI integration in nursing education requires more than increasing students’ AI knowledge. Educational strategies should simultaneously strengthen AI literacy and AI self-efficacy through structured instruction, guided practice, and opportunities to critically evaluate AI-generated information. Such approaches may better prepare nursing students to use GenAI safely, ethically, and effectively in future clinical practice. Future longitudinal studies are warranted to clarify the causal relationships among AI literacy, AI self-efficacy, and AI attitudes.

## 5. Limitations

Several limitations warrant consideration when evaluating these findings. First, because this study employed a cross-sectional design, it cannot establish temporal precedence, infer causality, or rule out alternative reciprocal pathways among AI literacy, AI self-efficacy, and AI attitudes. Longitudinal designs are required to clarify these directional trajectories over time. Second, because participants were recruited using convenience sampling from a single urban public community college, the generalizability of these findings may be limited to student populations with characteristics similar to those of the study cohort. Third, the use of self-reported questionnaires administered during a single survey session may introduce common method bias. Unmeasured participant characteristics may have influenced the observed associations. Future studies should incorporate relevant demographic and experiential variables as covariates. Fourth, participants with incomplete responses were excluded from the analysis. Consequently, the findings may be subject to nonresponse bias, which could limit the generalizability of the results. Furthermore, although validated instruments were used and observed total scores were analyzed, construct validity was not re-examined in the current sample. This should be considered when interpreting the findings.

Finally, a methodological consideration of this study concerns the differing levels of construct specificity across the measurement tools. While the AI Literacy Scale (AILS) and AI Self-Efficacy Scale (AISES) assess broad AI competencies, the GAI Attitude Scale (GAIAS) evaluates attitudes specifically toward GenAI. Given that GenAI represents a prominent form of AI currently integrated into student learning environments, this conceptual overlap may support the relevance of the measures used in the present study. Nevertheless, this should be considered when interpreting the findings.

## 6. Conclusions

This study demonstrates that AI literacy is positively associated with both AI self-efficacy and AI attitudes among prelicensure nursing students, with AI self-efficacy serving as a critical statistical mediator within this observational framework. As the primary outcome, AI attitudes are particularly relevant as a proximal predictor of students’ behavioral intentions to engage with GenAI. These empirical insights highlight the potential value of moving beyond informal exposure to GenAI by introducing structured AI education into nursing curricula. Guided hands-on practice with GenAI tools, case-based learning, simulation exercises, and faculty-supported feedback may provide students with opportunities to apply AI knowledge in clinically relevant contexts while developing confidence in its responsible use. Integrating these educational strategies may help nursing students develop AI literacy and AI self-efficacy concurrently while preparing them to critically evaluate when and how AI can appropriately support, rather than replace, professional nursing judgment.

However, given the cross-sectional design, these statistical associations do not establish direct causal mechanisms. Future longitudinal studies should evaluate these proposed pathways and determine whether targeted educational strategies produce sustained improvements in AI attitudes, AI self-efficacy, and subsequent GenAI adoption before widespread curricular integration is recommended.

## Figures and Tables

**Table 1:** Participants’ general characteristics (N = 100).

Variable	Category	n (%)/M ± SD	Range
Age		30.25 ± 7.84	19–48
Gender	Female	71 (71.0)	
	Male	27 (27.0)	
	Prefer not to say	2 (2.0)	
Semester	Semester 1	36 (36.0)	
	Semester 2	44 (44.0)	
	≥Semester 3 No response	9 (10.0) 11(11.0)	
Current Cumulative GPA	2.5–<3.0	6 (6.0)	
	3.0–<3.5	26 (26.0)	
	3.5–4.0 No response	63 (63.0) 5 (5.0)	
Computer Experience Level	Beginner	10 (10.0)	
	Intermediate	64 (64.0)	
	Advanced	23 (23.0)	
	No response	3 (3.0)	
Frequency of Generative AI Use for Academic Purposes	Never	4 (4.0)	
	Rarely (1–2/semester)	10 (10.0)	
	Occasionally (1–2/month)	18 (18.0)	
	Frequently (weekly)	27 (27.0)	
	Very frequently(multiple times/week) No response	39 (39.0) 2(2.0)	

Note. AI = artificial intelligence.

**Table 2:** Study variables and correlations between variables (n = 100).

Variables	*r* (*p*)		M ± SD	Range
	1	2		
1. AI Literacy	1		4.84 ± 0.80	1–7
2. AI Self-Efficacy	0.42 (<0.001)	1	4.90 ± 0.89	1–7
3. AI Attitudes	0.40 (<0.001)	0.42 (<0.001)	3.30 ± 0.49	1–5

Note. AI = Artificial Intelligence.

**Table 3:** Results of path analysis.

Hypotheses	Association	B	β	SE	C. R.	*p*	95% Bootstrap CI
H1	AI Literacy -> AI Self-efficacy	0.43	0.39	0.103	4.206	<0.001	[0.28, 0.64]
H2	AI Self-efficacy -> AI Attitudes	0.17	0.31	0.052	3.236	0.001	[0.06, 0.29]
H3	AI Literacy -> AI Attitudes	0.17	0.28	0.058	2.920	0.003	[0.06, 0.28]
H4	AI Literacy -> AI Self-efficacy -> AI Attitudes (Indirect)	0.07	0.12				[0.03, 0.15]

Note. AI = Artificial Intelligence; B = unstandardized estimate; β = standardized estimate. The 95% confidence intervals were obtained from bootstrap estimates (5000 resamples) and are based on unstandardized direct and indirect effects.

## Data Availability

The data presented in this study are available on request from the corresponding author due to privacy restrictions regarding participant data.

## Public Involvement Statement

No public involvement in any aspect of this research.

## Guidelines and Standards Statement

This manuscript was drafted in accordance with the Strengthening the Reporting of Observational Studies in Epidemiology (STROBE) Statement for cross-sectional studies [31].

## Abbreviations

The following abbreviations are used in this manuscript:

GenAI	Generative Artificial Intelligence
